# Novel Closure Technique With Double‐Balloon Endoscopy‐Guided Embolization by Gelatin Sponge and Clip Suturing for Postoperative Enterocutaneous Fistula With Bile Leakage

**DOI:** 10.1002/deo2.70287

**Published:** 2026-01-23

**Authors:** Yuki Ito, Hiroshi Yukimoto, Kohsaku Ohnishi, Takafumi Tanimoto, Motohiro Hirao, Yasuhiro Nakaya, Daisuke Takiuchi, Masanori Tsujie, Atsushi Hosui, Naoki Hiramatsu

**Affiliations:** ^1^ Department of Gastroenterology and Hepatology Osaka Rosai Hospital Sakai Osaka Japan; ^2^ Department of Diagnostic Radiology Osaka Rosai Hospital Sakai Osaka Japan; ^3^ Department of Gastroenterological Surgery Osaka Rosai Hospital Sakai Osaka Japan

**Keywords:** bile leakage, double‐balloon endoscopy, enterocutaneous fistula, fistula embolization, gelatin sponge

## Abstract

A 60‐year‐old female with abdominal pain and nausea was diagnosed with gallbladder cancer (cT3aN2M1, cStage IVB). Distant metastases had disappeared after 10 courses of chemotherapy, followed by conversion surgery consisting of subtotal stomach‐preserving pancreaticoduodenectomy, partial hepatectomy, and dissection of para‐aortic and regional lymph nodes. On postoperative day (POD) 3, additional drainage was performed from the median incision because of bile leakage (BL) from the choledochojejunostomy. The drain tube was removed on POD 89, and the patient was discharged. On the 13th day after discharge, BL was found from the median incision. Double‐balloon endoscopy‐guided endoscopic retrograde cholangiopancreatography was performed to investigate the cause of BL and decompress the bile duct. Although cholangiography revealed no obvious injury of the bile duct, endoscopic examination discovered a fistulous opening near the choledochojejunostomy, which was revealed to be the responsible lesion of the enterocutaneous fistula by contrast imaging. Closure of the enterocutaneous fistula was performed with a combination of embolization by gelatin sponge and endoscopic clip suturing. Rapid fistula closure was achieved after the procedure, and the patient was discharged without complications. This treatment method is considered a minimally invasive and effective therapeutic option for BL associated with postoperative enterocutaneous fistulas.

## 1 Introduction

Bile leakage (BL) is one of the complications of hepatobiliary surgery that can lead to infection and prolonged hospitalization, with an incidence of 3%–8% in pancreaticoduodenectomy [1]. Traditionally, percutaneous drainage or surgical reoperation has been performed to treat BL, but recent advances in endoscopic techniques make it possible to explore more minimally invasive and effective treatments. A less invasive treatment for BL is an urgent issue because surgical treatment of enterocutaneous leakage is particularly associated with high invasiveness (postoperative mortality rate 4.8% and complication rate 82.5%) [2]. Endoscopic treatments for enterocutaneous fistulas include closure with Over‐The‐Scope Clips (OTSC), clip suturing, and injection of fibrin glue. Percutaneous treatments with embolic materials such as gelatin sponge [３] and *n*‐butyl‐2‐cyanoacrylate [４] for fistula closure have been reported. This case report describes successful treatment with double‐balloon endoscopy‐guided embolization by gelatin sponge and clip suturing for an enterocutaneous fistula complicated by BL after subtotal stomach‐preserving pancreaticoduodenectomy (SSPPD).

## 2 Case Report

**FIGURE 1:**
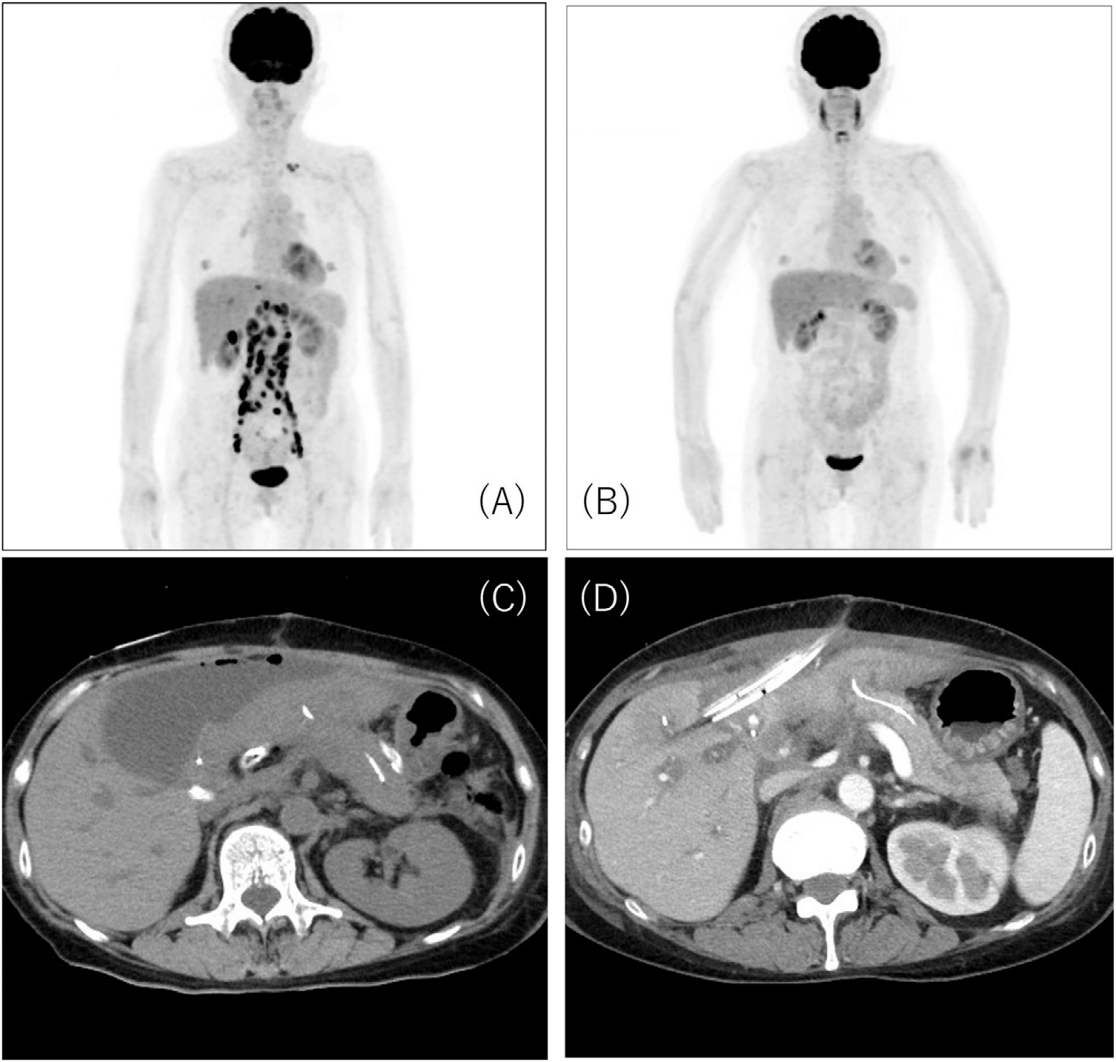
Imaging findings of gallbladder cancer. (A) Positron emission tomography‐computed tomography (PET‐CT) before chemotherapy. Distant metastases were observed. (B) PET‐CT after chemotherapy. Distant metastases disappeared. (C) A retentive cavity was identified on the ventral side of the elevated jejunum. (D) Additional drainage was performed at the retentive cavity through the midline incision.

A 60‐year‐old female visited our hospital because of abdominal pain and nausea. Detailed examination, including abdominal ultrasound, contrast‐enhanced computed tomography (CT), and positron emission tomography (PET)‐CT, revealed gallbladder cancer with metastases to lymph nodes and liver (Figure 1A). Based on UICC 8th edition criteria, she was diagnosed with gallbladder cancer (cT3aN2M1, cStage IVB), and gemcitabine/cisplatin/durvalumab (GCD) therapy was initiated.

After 10 courses of GCD therapy, PET‐CT showed the disappearance of abnormal uptake from distant metastases (Figure 1B), and then conversion surgery was performed. All rapid histological examinations of para‐aortic lymph nodes during surgery were negative, followed by conversion surgery consisting of SSPPD, partial hepatectomy, and dissection of para‐aortic and regional lymph nodes.

**FIGURE 2:**
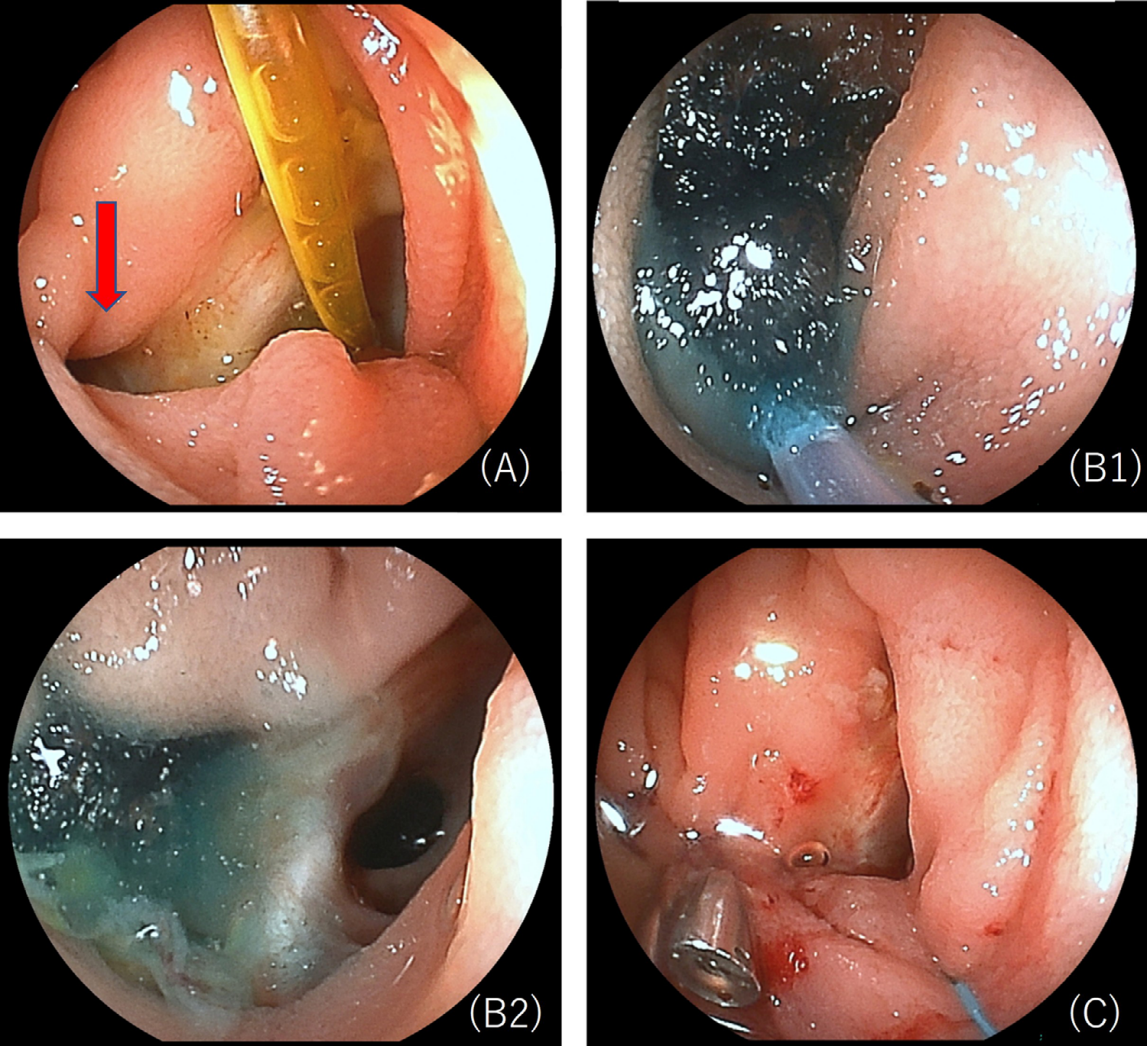
Double‐balloon endoscopy (DBE) images. (A) A postoperative lost stent was observed at the choledochojejunostomy anastomosis, and an enterocutaneous fistula (red arrow) was identified in its vicinity. (B1, B2) Embolic material consisting of a gelatin sponge, contrast agent, and a small amount of indigo carmine was injected into the fistula site through the catheter. (C) Suturing was added to the fistula site using endoscopic clips.

**FIGURE 3:**
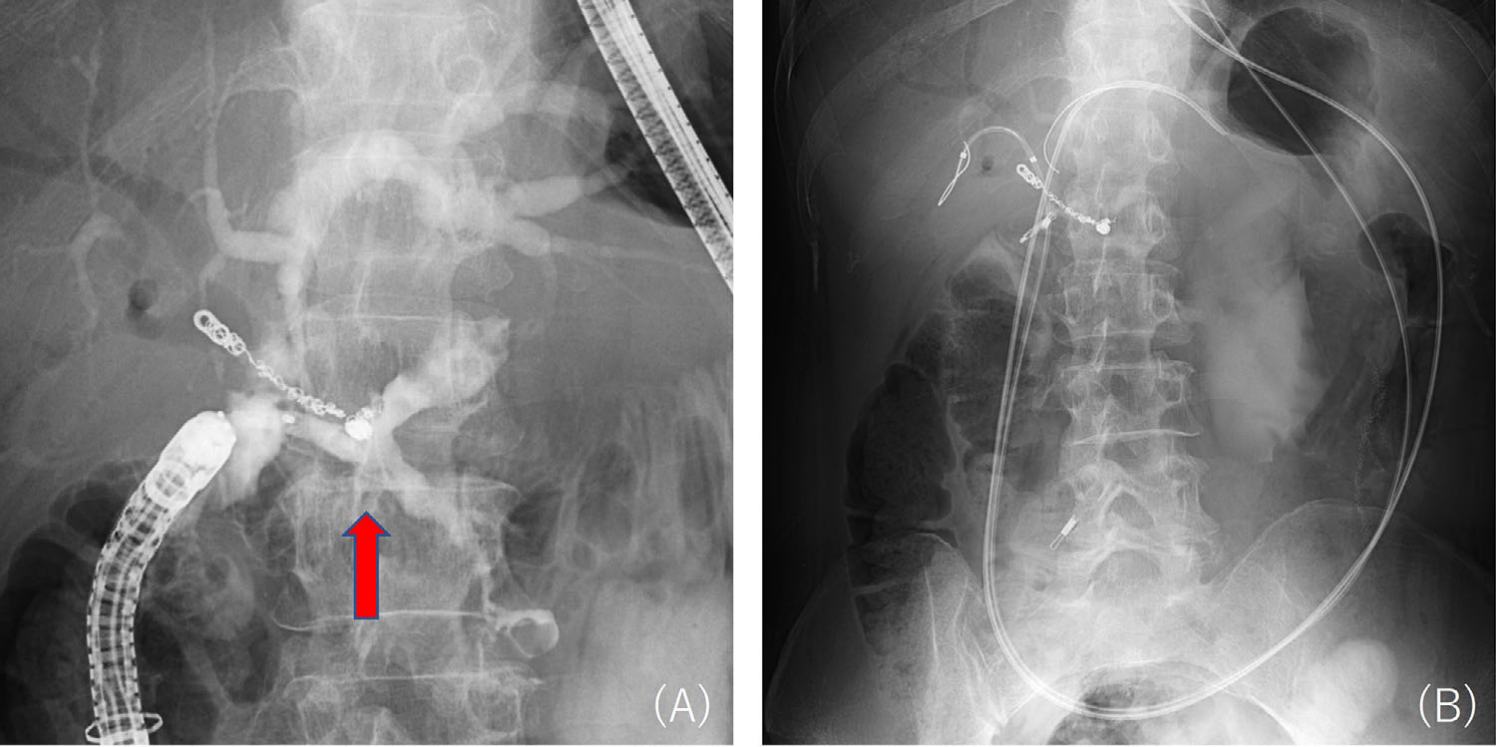
Fluoroscopic images during double‐balloon endoscopy (DBE). (A) Cholangiography revealed an enterocutaneous fistula (red arrow). (B) Endoscopic nasobiliary drainage (ENBD) tubes were placed in the left and right bile ducts.

On postoperative day (POD) 3, the bilirubin level in the drain fluid near the choledochojejunostomy was high (drain fluid bilirubin 24.7 mg/dL, serum bilirubin 0.6 mg/dL), leading to a diagnosis of BL based on the International Study Group of Liver Surgery (ISGLS) definition [5]. On POD 10–12, body temperature rose to 40°C, and BL from the median incision and localized peritonitis were observed (Figure 1C). Additional drainage was performed, and symptoms improved (Figure 1D). The drain tube was removed on POD 89, and the patient was discharged. On day 13 after discharge, the patient developed a high fever with drainage from the median incision (drainage fluid bilirubin 11 mg/dL, serum bilirubin 0.4 mg/dL), requiring readmission. Despite improvement in fever and inflammatory markers with conservative treatment with fasting and antibiotics, BL persisted at approximately 100–150 mL/day. Double‐balloon endoscopy‐guided endoscopic retrograde cholangiopancreatography (DBE‐ERCP) was performed to investigate the cause of BL and decompress the bile duct with an upper DBE scope (EI‐580BT, Fujifilm, Tokyo, Japan). Although cholangiography showed no obvious injury of the bile duct, a 5‐mm fistulous opening was discovered near the choledochojejunostomy (Figure 2A). Injection of contrast agent through this site using a catheter (MTW ERCP catheter; MTW Endoscopie, Wesel, Germany) showed contrast extravasation outside the bowel. Consistent with the previously retained drain course, the contrast was discharged to the skin fistula (Figure 3A), confirming the responsible enterocutaneous fistula based on the CT findings. This treatment strategy was determined to be embolization by gelatin sponge combined with clip suturing for closure of the enterocutaneous fistula. Our institutional ethics committee determined that formal ethical review was not required, as this treatment represented a variation of the well‐established percutaneous embolization technique for enterocutaneous fistulas. Written informed consent was obtained from the patient after discussing the procedure, potential complications, and alternative treatment options. An embolic material was created using one gelatin sponge sheet (Sponzel; LTL Pharma Co., Ltd., Tokyo, Japan), contrast agent (Iopamidol 300 mg/mL, Iopamiro 300 mg/mL; Bayer Schering Pharma, Osaka, Japan), and a small amount of indigo carmine. This embolic material was injected into the fistula site through the catheter (Figure 2B1,B2), and reliable retention of the embolic material was confirmed under fluoroscopic and endoscopic guidance. Subsequently, suturing was added to the fistula site using endoscopic clips (MANTIS Clip; Boston Scientific Japan Co., Ltd., Tokyo, Japan) (Figure 2C). Finally, endoscopic nasobiliary drainage (ENBD) tubes were placed in the left and right bile ducts (Figure 3B). After the procedure, BL rapidly decreased (less than approximately 10 mL/day), and finally, on day 7 after the procedure, the cholangiography showed no visualization of the fistula site, confirming that it was completely closed. After ENBD tube removal, there was no increase in BL, and the patient was discharged on day 14 after the procedure. No fistula recanalization was observed at 2 months of follow‐up.

## 3 Discussion

This case is the first report using DBE‐guided embolization by gelatin sponge combined with clip suturing for postoperative enterocutaneous fistula with BL. According to ISGLS classification, this Grade B BL typically requires additional drainage and prolonged hospitalization [6]; minimally invasive alternatives are critically important. The successful treatment for this complex condition with a novel combination approach offers significant clinical implications for managing similar challenging cases. The rationale for treatment selection in this case was to avoid surgical reoperation from the view point of invasiveness. A percutaneous approach was not feasible because the drain tube targeting the fistula site had already been removed, and an obvious fistula or cavity was not visible on the contrast‐enhanced CT scan performed at admission. Endoscopic treatment was selected because of its low invasiveness and low risk of serious complications. The DBE procedure enabled us to safely observe postoperative reconstructed bowel and to elucidate the cause of BL, leading to the discovery of the fistula. The endoscopic approach enables accurate diagnosis of fistula location and size before treatment, allowing safer and more reliable therapy even when traditional percutaneous access is challenging. In this case, cholangiography made it possible to diagnose that an enterocutaneous fistula was near the choledochojejunostomy, which caused BL. Conventional endoscopic biliary drainage alone would be difficult or lead to prolonged hospitalization. Although OTSC was considered, it was not routinely available at our institution, and deploying it under DBE guidance is technically challenging. Additionally, the close proximity of the fistula to the choledochojejunostomy raised concerns about potential anastomotic obstruction or stenosis. Since endoscopic clips alone raised concerns about fistula recurrence [5], combination therapy with gelatin sponge and endoscopic clips was selected for this case.

Gelatin sponge has beneficial effects to physically occlude and to promote tissue repair through the inflammatory response. It is absorbed in the body within 4–6 weeks, and has excellent safety with low risk of long‐term foreign body reactions. The combination of leak pressure reduction through clip suturing and tract pressure increases through embolization by gelatin sponge is a theoretically appropriate treatment for this pathology. The embolic material, which was visible under both fluoroscopic and endoscopic guidance, enabled precise identification and treatment of the fistula tract.

The efficacy of embolization by gelatin sponge via percutaneous approach for closing enterocutaneous and biliary fistulas has been reported [3, 7], suggesting that endoscopic delivery, as documented in this case, may represent a feasible and effective treatment option. As for other embolic materials, percutaneous embolization with *n*‐butyl‐2‐cyanoacrylate or cyanoacrylate and lipiodol mixture provided 100% clinical success rate (11/11 cases), (7/7 cases), respectively [4, 8]. Various embolic materials exert fistula closure effects through different mechanisms [9], and embolic materials used in percutaneous approaches are likely to be applicable to endoscopic approaches as well.

Indications for this treatment method include endoscopically accessible sites, fistulas amenable to closure with endoscopic clips, and well‐controlled infection.

The feasibility of clip closure depends largely on operator expertise and technical factors. Adequate infection control is essential, as introducing foreign material such as gelatin sponge in the setting of uncontrolled infection may exacerbate the infection. In this case, infection was well‐controlled prior to the procedure, satisfying all criteria for endoscopic gelatin sponge embolization.

No procedure‐related complications were observed in this case, and the combination of endoscopic and fluoroscopic guidance may provide more precise embolization.

In conclusion, endoscopic embolization by gelatin sponge combined with clip suturing for postoperative BL is a promising, minimally invasive, and effective treatment option similar to percutaneous approaches, representing a valuable treatment selection that can avoid surgical reoperation. However, larger case series and comparative studies are further needed to establish definitive efficacy and safety.

## Author Contributions

**Yuki Ito** and **Hiroshi Yukimoto** contributed to this work as co‐first authors.**Yuki Ito**: endoscopic procedures, clinical management (outpatient and inpatient care), writing – original draft, and writing – review and editing. **Hiroshi Yukimoto**: interventional radiology procedures (fistula embolization), imaging data acquisition and analysis, literature research, writing – original draft, and writing – review and editing. **Kohsaku Ohnishi**: treatment planning and coordination, endoscopic procedures support, clinical management, and writing – review and editing. **Takafumi Tanimoto**: clinical management, endoscopic procedures support, and writing – review and editing. **Motohiro Hirao**: clinical management, endoscopic procedures support, and writing – review and editing. **Yasuhiro Nakaya**: Overall supervision of interventional radiology procedures, imaging guidance, and writing – review and editing. **Daisuke Takiuchi**: Initial surgical procedure (SSPPD), postoperative surgical management, surgical expertise consultation, and writing – review and editing.**Masanori Tsujie**: Initial surgical procedure (SSPPD), postoperative surgical management, surgical expertise consultation, and writing – review and editing. **Atsushi Hosui**: Overall clinical supervision, final treatment decisions, and writing – review and editing; co‐guarantor of the entire case. report. **Naoki Hiramatsu**: Overall clinical supervision and writing – review and editing; guarantor of the entire case report.

## Conflicts of Interest

The authors declare no conflicts of interest.

## Funding

None.

## Ethics Statement

Informed consent for publication, including radiologic and endoscopic images, was obtained from the patient.
